# Care managers can be useful for patients with depression but their role must be clear: a qualitative study of GPs’ experiences

**DOI:** 10.1080/02813432.2019.1639897

**Published:** 2019-07-09

**Authors:** Sandra af Winklerfelt Hammarberg, Dominique Hange, Malin André, Camilla Udo, Irene Svenningsson, Cecilia Björkelund, Eva-Lisa Petersson, Jeanette Westman

**Affiliations:** a Division of Family Medicine and Primary Care, Department of Neurobiology, Care Sciences and Society, Karolinska Institutet, Stockholm, Sweden;; b Academic Primary Health Care Centre, Stockholm County Council, Stockholm, Sweden;; c Department of Public Health and Community Medicine, Primary Health Care, Institute of Medicine, The Sahlgrenska Academy, University of Gothenburg, Gothenburg, Sweden;; d Department of Public Health and Caring Sciences – Family Medicine and Preventive Medicine, Uppsala University, Uppsala, Sweden;; e School of Education, Health and Social Studies, Dalarna University, Falun, Sweden;; f CKF, Center for Clinical Research Dalarna, Falun, Sweden;; g Närhälsan Research and Development Primary Health Care, Region Västra Götaland, Gothenburg, Sweden;; h Division of Nursing, Department of Neurobiology, Care Sciences and Society, Karolinska Institutet, Stockholm, Sweden

**Keywords:** Primary care, family medicine, nursing interventions, coordinated care, psychiatry, patient care continuity, collaborative care

## Abstract

**Objective:** Explore general practitioners’ (GPs’) views on and experiences of working with care managers for patients treated for depression in primary care settings. Care managers are specially trained health care professionals, often specialist nurses, who coordinate care for patients with chronic diseases.

**Design:** Qualitative content analysis of five focus-group discussions.

**Setting:** Primary health care centers in the Region of Västra Götaland and Dalarna County, Sweden.

**Subjects:** 29 GPs.

**Main outcome measures:** GPs’ views and experiences of care managers for patients with depression.

**Results:** GPs expressed a broad variety of views and experiences. Care managers could *ensure care quality while freeing GPs from case management* by providing support for patients and security and relief for GPs and by coordinating patient care. GPs could also express *concern about role overlap*; specifically, that GPs are already care managers, that too many caregivers disrupt patient contact, and that the roles of care managers and psychotherapists seem to compete. GPs thought *care managers should be assigned to patients who need them the most* (e.g. patients with life difficulties or severe mental health problems). They also found that *transition to a chronic care model required change*, including alterations in the way GPs worked and changes that made depression treatment more like treatment for other chronic diseases.

**Conclusion:** GPs have varied experiences of care managers. As a complementary part of the primary health care team, care managers can be useful for patients with depression, but team members’ roles must be clear.

**KEY POINTS**
- A growing number of primary health care centers are introducing care managers for patients with depression, but knowledge about GPs’ experiences of this kind of collaborative care is limited.
- GPs find that care managers provide support for patients and security and relief for GPs.
- GPs are concerned about potential role overlap and desire greater latitude in deciding which patients can be assigned a care manager.
- GPs think depression can be treated using a chronic care model that includes care managers but that adjusting to the new way of working will take time.

## Introduction

Primary health care plays a crucial role in treating patients with chronic diseases [1,2], which can include depression [2–4], one of the most common mental disorders and a major source of disability worldwide [5]. In Sweden, more than 70% of patients with depression receive treatment in primary care, and 65% of all prescriptions for antidepressants are written there [6].

Primary care has increasingly turned to chronic disease management models to improve care quality and continuity for patients with chronic diseases [1]. Inspired by these models, collaborative care interventions seek to improve patient care [7], for example by facilitating patients’ self-management efforts and adherence to treatment. Additionally, they promote communication, coordination, and involvement of health care providers in and outside primary care [8]. Care managers are a crucial component of many collaborative care interventions. These specially trained health care professionals, often specialist nurses, coordinate care for patients [9,10] and provide follow-up to patients and feedback to physicians [10]. Care managers have been used to manage care for patients with diabetes [11–13], other single chronic somatic diseases [9,14], and multiple chronic somatic diseases [1]. Care manager programs for somatic diseases have been implemented in several countries [9,11,12,14]. In the United Kingdom [4–6,15], United States [15–23], and the Netherlands [24] care managers have also been successfully used for primary health care patients with depression. However, employing care managers to support patients with depression is new in Swedish primary health care [25].

Previous care manager projects for patients with depression were undertaken because patient access and follow-up were in need of improvement [26–29]. Sweden also experiences problems with access and follow-up in depression care. However, the organization of primary care in Sweden differs from that of countries where previous studies have taken place, including the United States [26], United Kingdom [29], and Germany [27]. In Sweden’s government-funded, decentralized health care system, public and private primary health care centers answer to county councils or regional authorities. General practitioners (GPs) and nurses are salaried employees of a center, which may also employ other kinds of health care professionals (e.g. psychotherapists).

This qualitative study was conducted during a randomized controlled trial (RCT) that tested the clinical effectiveness of care managers for patients with depression in Swedish primary health care. The RCT was conducted by our research group in the region of Västra Götaland and in Dalarna County between 2014 and 2016 [8]. We found that patients with care managers (the intervention group) recovered from depression symptoms and returned to work faster than patients who received usual care (the control group) [8]. The program was also cost-effective [30]. A subsequent qualitative study of nurses’ experiences of working as care managers found that the care managers viewed themselves as a safety net for patients [31]. Additionally, the care managers thought they increased the accessibility and continuity of care.

Qualitative studies from outside Sweden have found that GPs think of care managers as a valuable addition to the collaborative care team for patients with depression [32,33]. GPs also say that collaboration is facilitated when they share the same patient record system and the same workplace with the care managers, as this makes communication easier [32]. Moreover, GPs feel that patients with complex and more severe problems (e.g. complex mental health problems, comorbidity, and psychosocial problems) have the greatest need for care managers [32]. However, we also wanted to know how GPs in Sweden experienced working with care managers for patients with depression.

### Aim

We conducted a qualitative study to explore GPs’ views on and experiences of working with care managers for patients treated for depression in primary care settings.

## Material and methods

### Study design

In this qualitative study, we conducted focus-group interviews with GPs in two geographical areas in Sweden. Focus groups were used in the expectation that participants’ shared experiences of working with care managers would enrich discussions [34]. Qualitative content analysis was used to analyze transcripts of the interviews [35,36].

### Setting and participants

This study took place at primary health care centers in the region of Västra Götaland (population 1.7 million) and in Dalarna County (population 290,000), the two regions in Sweden that were participating in our RCT on the use of care managers for patients with depression [8]. A total of 23 centers participated in the RCT, and five centers still recruiting patients to the RCT took part in the focus-group discussions. They were located in urban and rural areas and neighborhoods of varying socioeconomic status. GPs at the primary health care centers were invited via email to participate in the focus groups. We held five focus groups, one at each health care center, with 29 participants between the ages of 28 and 66 years, 16 men and 13 women. All were GPs or physicians undergoing the 5 years of specialist training in primary care needed to become a GP in Sweden. Participants’ working experience ranged from a few years as medical residents to over 20 years as GPs.

### Randomized controlled trial of care managers for patients with depression in primary health care

This qualitative study was conducted during the RCT that tested the clinical effectiveness of care managers. In the RCT, primary health care center patients diagnosed with mild and moderate depression less than 1 month previously, who were identified by their GPs, were offered the opportunity to meet with a care manager to set up an individualized care plan. GPs had diagnosed depression on the basis of ICD-10 criteria (codes F32 and F33) [37]. Depression severity was determined with the Montgomery-Asberg Depression Rating Scale – Self assessment (MADRS-S) instrument [38]. The intervention consisted of continuous contact between care manager and patient, a structured management plan and six structured telephone calls during the 12 weeks following referral. In these calls, the care managers assessed and monitored patients’ depressive symptoms via the Montgomery–Asberg Depression Rating Scale (MADRS) (including suicidal ideation), encouraged treatment adherence, supported behavioral activation, and coordinated care. The care manager maintained close communication with the patient’s GP and planned collaborative care at the center. The care manager’s primary responsibility was to regularly follow up and monitor depression and inform the GP if the patient did not respond adequately to treatment [8].

### The focus groups

The focus groups took place during regular opening hours at the primary health care center where the GPs worked. The potential participants were familiar with the research group, as the primary health care center was participating in the RCT. Four weeks before the planned start of the interviews, a letter describing the focus-group study was sent to the selected centers. At the beginning of each focus group, the moderator provided verbal information about the study and explained that participation was voluntary and that the information participants provided would be treated confidentially. Participants then gave both verbal and written consent prior to the start of the focus-group interviews. Five of the authors (SWH, DH, MA, CU, and JW) conducted the focus groups. At each focus group, one acted as moderator and another as observer. The moderators had experience conducting focus groups and used a topic guide with open questions that was developed for the study. Each focus-group discussion took about 60 min, was audio recorded, and was transcribed verbatim prior to analysis.

### Analysis

To investigate the experiences of the GPs, the transcribed focus-group discussions were analyzed with qualitative content analysis as described by Graneheim and Lundman [35,36]. Qualitative content analysis enables a systematic analysis of variations in texts [35,39]. An inductive approach was used [36,39]. Three of the authors conducted the initial data coding. SWH was the main coder. To start the process, the researchers read the text several times to get a sense of the whole and then discussed their first impressions. Using a spreadsheet, they then divided the text into meaning units. The meaning units relevant to the aim were shortened to condensed meaning units, which remained close to the text in meaning. The condensed meaning units were labeled with codes that expressed their content. The codes were then sorted on the basis of similarities and differences and grouped into subcategories and categories (Table 1). To help ensure trustworthiness, all researchers reflected on and discussed the emerging categories and their potential meaning until they reached consensus.

**Table 1.** Overview of subcategories and categories.

Code	Subcategory	Category
Someone who calls and checks on patients	Care managers provide support for patients	Care managers ensure care quality while freeing GPs from case management
A relief that the care manager maintains contact with patients	Care managers provide security and relief for GPs	
Coordinator provides patient follow-up, detects risk	Care managers ensure coordinated care for patients	
I myself have to follow up	GPs are already care managers	Concern about role overlap
Personal contact is important	Too many caregivers disrupt patient contact	
If resources are limited, we would rather prioritize a psychotherapist	The roles of care managers and psychotherapists seem to compete	
Help needed for problems other than depression	Patients with life difficulties need care managers	Care managers should be assigned to patients who need them the most
Sicker patients have a greater needs	Patients with severe mental health problems have a greater need for care managers	
You have to get used to working with care managers.	Care managers change the way GPs work.	Transition to a chronic care model for depression requires change
Care managers make treatment for depression more similar to treatment for other chronic diseases	Depression treatment becomes more like other chronic disease treatment	

All the researchers had worked in primary health care with patients who had depression. However, none had previously worked with care managers. The researchers came from different primary health care professions and geographical areas in Sweden. Four were GPs, two were district nurses, and two had other health care professions. SWH was a doctoral student, and the others had PhDs.

## Results

Four categories of experiences emerged from the analysis: *Care managers ensure care quality while freeing GPs from case management*, *Concern about role overlap*, *Care managers should be assigned to patients who need them the most*, and *Transition to a chronic care model requires change*.

### Care managers ensure care quality while freeing GPs from case management

This category described GPs’ perception that care managers could lighten GPs’ workloads while maintaining care quality.

#### Providing support for patients

Some GPs thought patients were pleased to have someone to contact who cared about them. According to the GPs, it was important for patients to feel taken care of and to have ongoing support, as this could improve adherence to treatment. It could also increase patients’ motivation for behavioral change, including physical activity.

And partly … yes but partly as a kind of support, and then she’s also followed up with her–—these rating scales, so that she’s been able to tell if the patient feels better or worse and follow the course, and it’s in those cases that she’s been like able to signal, that yes, now it’s going in the right direction or now it’s going in the wrong direction, especially if it’s going in the wrong direction. (Focus group 2, line 164)

#### Providing security and relief for GPs

Many GPs thought it was a relief to have a care manager because it gave them something to offer patients. They thought care managers provided added value as an extra counseling resource. The care manager was also an easily accessible person with whom the GPs could discuss patient assessment and follow-up. The GP could quickly provide an appointment with the care manager and felt safe knowing that the care manager would follow up, for example via regular use of rating scales. The care manager could thus also let the GP know if the patient’s condition deteriorated. Some GPs did not think they had the opportunity to give patients the support and continuity they needed. These GPs described how care managers enabled them to refine their role as a doctor and focus on patients’ medication, physical problems and tests, and in some cases, sick leave. GPs were able to do less telephone follow-up because they felt confident that the care manager had control of the situation.

So it feels like a big relief that you can hand over to her, just those … it’s important for patients that someone just gets in touch so to speak. (Focus group 2, line 281)

#### Ensuring coordinated care for patients

According to GPs, care managers’ contributions to coordinated care included bringing increased attention to patients with depression, thus improving the overall care the patients received. Moreover, care managers’ nursing skills complemented the GPs’ skills. For example, the nurses had experience in patient assessment by telephone, including adherence to medication.

Care managers could facilitate teamwork and communication about patients, especially when they worked at the same primary health care center as the GP. Documenting their work in the same patient records and being able to talk even during breaks made it easier to collaborate with the care manager. When they worked at the same center and on the same team as the care managers, GPs could also see whether the care managers had characteristics that made them trustworthy. These included thoroughness, dependability, and good communication skills.

I would see it as a coordinator, another voice that … takes care of the patient, that sees to it that the depression doesn’t escalate, that a situation that demands radically different treatment doesn’t develop, threat of suicide or something else like that. (Focus group 2, line 152)

### Concern about role overlap

GPs expressed several concerns about ways in which the care manager’s role might conflict with those of other health care professionals, including GPs and psychotherapists.

#### GPs are already care managers

GPs sometimes felt they did not need care managers and explained that they had not changed the way they worked after care managers were introduced. They still thought it was their responsibility to follow up patients and that they had the competence to follow up treatment, medication, test results, and sick leave. In particular, GPs at well-staffed primary health care centers where patients could easily meet their own GP, even for follow-up visits, did not feel the need to have another person act as the patient’s care manager. Some expressed concern about losing the supportive component of their role.

Then it’s like I probably know the patient best so I’m actually the care manager, anyway I think, me as the doctor. (Focus group 4, line 151)

To be honest, I don’t think we have the need in the same way, because as I said, so far, GP accessibility has been so good here that the patient can even get in contact with their doctor the same day. We can even offer a visit, extra visit the same day. (Focus group 4, line 163)

#### Too many caregivers disrupt patient contact

GPs could be doubtful about handing patients over to care managers. The GPs had built relationships with the patients, who had sometimes told them about very personal matters. It was then uncomfortable to hand patients over to someone else for follow-up – a person who did not know the patients. Moreover, some GPs had met patients with depression who found it difficult to manage numerous caregivers. They explained that this was a reason many patients had declined to participate in the study.

Often you establish some kind of contact. That contact is very important with regard to this type of personal contact. Then it’s very strange, I think, to pass it on to another person. (Focus group 3, line 173)

They drop out because they experience too many people around them, and maybe they’ve told us something hard and don’t want to tell a lot of people about it. (Focus group 5, line 242)

#### The roles of care managers and psychotherapists seem to compete

Another concern was that having care managers could take time and money from other priorities. Some GPs expressed a preference for easier access to a psychotherapist rather than access to a care manager. ‘In the best of all possible worlds it would in fact be good to have a therapist here,’ said one GP (Focus group 1, line 794). However, GPs said patients sometimes had to wait a long time for psychotherapy and that care managers could keep in contact with patients during this waiting time. Moreover, care managers could play a role in long-term follow-up. GPs could feel more trust in care managers who had training in fields such as cognitive behavioral therapy (CBT), substance use, and rehabilitation (i.e. those trained in counseling patients returning to work after sick leave) than in those who did not have such training.

Today we have a full-time CBT [therapist] who is fully booked, and then we add a part-time nurse at over 30,000 SEK (3000 Euros) a month, who can afford that? (Focus group 4, line 333)

### Care managers should be assigned to patients who need them the most

GPs expressed a desire for more flexible access to care managers than they had in the RCT, as this would help them support the full spectrum of patients they met in clinical practice.

#### Patients with life difficulties need care managers

GPs described meeting patients whose problems, in the opinion of the GPs, were not primarily medical. Instead, they thought the problems were complex and of a social nature and were concerned that the problems would be unnecessarily medicalized. Examples of such life difficulties included unemployment, loneliness, everyday stress, and problems with work and/or relationships. GPs thought that these patients might not benefit from medical treatment or even psychotherapy. However, someone like a care manager could provide them with a feeling of security and continuity and GPs with relief.

Good if you impartially ask yourself the question ‘Does the patient really need to see a doctor?’ when the patient contacts primary health care. Problems can be medicalized unnecessarily. (Focus group 2, line 295)

#### Patients with severe mental health problems have a greater need for care managers

The spectrum of patients that GPs met in everyday clinical practice also included patients with more severe, recurring depression or multiple, complex problems. In addition to having life difficulties, such patients could have mood and anxiety disorders, adjustment disorders, substance use disorders, or personality disorders. Some GPs thought it was wrong to exclude patients with more severe or complex mental health problems, as this meant that those with milder problems (uncomplicated depression) received more care than those with greater need.

Yes, but I believe that the one who had a high MADRS-S score [severe depression] … clinically that it was the right level [to be assigned a care manager]. (Focus group 1, line 516)

Because I think it’s another group of patients that doesn’t … that weighs down the telephone and the doctor a lot, it’s people … before you called them a little anxious … before it was called anxiety or GAD or a little personality disorder or borderline. Now, I don’t know what it’s called today, but … I was thinking … because just there you should maybe have a telephone … a nurse … instead of calling, waiting for them to call, because there’s always something new. (Focus group 2, line 758)

### Transition to a chronic care model for depression requires change

GPs described how care managers changed care for patients with depression and could feel that the change required some adjustment.

#### Care managers change the way GPs work

GPs could feel that it was challenging to get used to working with care managers. It meant becoming accustomed to new working habits, which was not always easy. One GP said that ‘Above all, you have to get used to using them. You’re used to one way of working that’s the norm, then that you do it in this [other] way’ (Focus group 3, line 383).

#### Depression treatment becomes more like other chronic disease treatment

GPs thought care managers helped make depression care more structured and standardized, and thus more similar to care for other common chronic diseases.

This is a huge group of patients because psychiatry in primary health care [is] enormous and growing, and those patients should also be cared for. We have specialized personnel for high blood pressure, for diabetes, for COPD at the health care center. It’s clear we should have that for this, too, as it’s become a big part of our mission. (Focus group 3, line 488)

## Discussion

### Main findings

Overall, GPs thought care managers could be useful for patients with depression, but their role must be clear. On the one hand, care managers could ensure care quality while reducing GPs’ workloads. On the other, the GPs had to adjust to working with a new care model. GPs were concerned about potential role overlap and wanted more freedom to choose which patients received a care manager.

The finding that care managers provide support for patients and security and relief for GPs is echoed in the findings of other studies [27,33,40]. In one of those studies, the researchers found, as we did, that care managers’ regular contact with patients and feedback to GPs were important to these positive experiences [27]. Similarly, in a survey of GPs who had participated in a collaborative care intervention for patients with late-life depression, the GPs pointed to active follow-up, monitoring, and information to the GPs as among the most positive aspects of the intervention [40].

At least one previous study has shown that GPs switch between biomedical and psychosocial perspectives depending on whether they characterize a patient’s problem as somatic or psychosocial [41]. In our study, some GPs thought care managers were unnecessary because the GPs saw themselves as care managers. We reason that these could be GPs who were adopting a psychosocial and holistic view of their role in caring for patients with depression. Moreover, the GPs in the current study who thought of themselves as care managers were concerned that care managers could disrupt doctor-patient relationships and burden patients with too many health care contacts. GPs in a German study also expressed the feeling that the care managers’ role overlapped with the GPs’ traditional role [27].

Care managers are intended as a complement to existing depression treatment. Their work is not meant to overlap with or replace that of psychotherapists or any other category of health care professional. However, in both our study and the German study [27], GPs seemed concerned about potential overlap between the role of care managers and the role of psychotherapists. In our study, some of these concerns could have resulted from confusion about the role of care managers. GPs received an introduction to the care manager model at the beginning of the RCT, but it may not have been enough. It is possible that jointly training all GPs and care managers prior to introducing the new role at a primary health care center would clarify GPs’ understanding of care managers, reducing concerns about overlapping roles and facilitating collaborative care.

Some GPs said they would rather have psychotherapists than care managers. This may be because cognitive behavioral therapy is a first-line treatment for depression [6], but not enough psychotherapists are available at health care centers to meet treatment needs. GPs also thought, however, that care managers could be helpful in the absence of access to psychotherapists; for example, for patients waiting in line or those who needed long-term follow-up.

GPs were also concerned that care managers were not assigned to the patients who needed them the most. In part, GPs’ concerns reflected the overall societal discussion of the problem of medicalizing everyday life difficulties [42]. GPs thought that a care manager’s follow-up and support might be all that some patients need and could thus minimize the risk of medicalization. Additionally, they wanted the freedom to refer patients with more chronic and severe mental health problems to care managers. An example could be patients with generalized anxiety disorder, who use more health care services than those without the disorder [43]. These findings are similar to those of previous studies, where GPs also expressed the wish for greater autonomy in deciding which patients they could refer to care managers [27,32].

The role of care managers is typically defined as part of a collaborative chronic care model [10]. Chronic care models presuppose the existence and implementation of clear, evidence-based guidelines [9]. In Swedish primary care, guidelines for depression [6] remain less fully implemented than certain other guidelines, such as those for cardiac and diabetes care. This incomplete implementation may help explain why GPs have varying views of the role of care managers in care for patients with depression. They may simply not have clear expectations of structured depression care. Additionally, we provided the GPs in our study with limited information on how to use care managers, and this could help explain why GPs had varying views of the role of care managers. In similar interventions in the future, we recommend training care managers, GPs, and psychotherapists as a team.

Our findings also suggest that GPs recognized the value of working together as a team with the care manager, with or without a psychotherapist. For example, the GPs in our study emphasized the importance of the care manager’s nursing and/or therapeutic competence, which they felt provided added value. Moreover, GPs viewed it as important to work at the same health care center as the care manager, as this facilitated team communication. They could also express doubts about the value of care managers if they experienced the care manager as part of a parallel process rather than part of the team. Similarly, other qualitative studies have found that GPs find it less than ideal when care managers work parallel to rather than in health care teams [27].

Much of the variation in GPs’ experiences of care managers seemed related to their differing perceptions of their roles as GPs, especially their role in treating patients with mental health problems. Variation also seemed to emerge from the way the GPs perceived conditions at their primary health care centers; for example, access to and continuity of care. Thus, GPs’ ideas of their role and situation prior to the introduction of care managers appear to have colored their view of this new role in the primary health care team.

As the GPs noted, change is not always easy, and transition to the new care model required adjustment. According to the theory of loss aversion, the subjective weight of a loss is heavier than the subjective weight of an equal gain [44]. Loss aversion could help explain the variation in GPs’ experiences of care managers, including the strength of some GPs’ concerns about role overlap. They might be weighing an expected role-related loss more heavily than potential gains related to introducing care managers. Aversion to loss might also help explain why the GPs’ experiences of care managers were more mixed than the experiences of the care managers themselves. The care managers described their new role as adding value to the existing team at their center by providing follow-up, support, and a safety net for patients, as well as increasing accessibility and continuity of care [31].

### Strengths and limitations of the study

The use of focus-group discussions to gather data strengthened the credibility of the current study, as focus groups are useful for exploring experiences. They have the advantage of promoting rich discussions [34], which may also have been facilitated by the participation of men and women and of people who had been working as GPs for varying lengths of time. A potential weakness was that the head of the primary health care center was present at two focus-group discussions, which may have influenced what was said. A strength was the variation in participating primary health care centers: focus groups were conducted in the northern and western regions of Sweden, in rural and urban areas, and at privately and publically run centers. This may strengthen the transferability of the results.

A further strength of the study was the use of qualitative content analysis, a method that can be inductive and is well-suited for exploring experiences [45]. In some qualitative content analyses, researchers identify themes, whereas in others, the analysis leads only to categories. In this analysis, we identified categories but no overarching theme that connected the categories.

The researchers’ prior knowledge was a potential strength, as they came to the study with an understanding of depression and of primary health care. However, it could also have been a weakness, as researchers’ experiences and views can affect focus-group discussions and analyses. To mitigate the influence of bias, the same two open questions guided all focus-group discussions, and in addition to the moderator, an observer was always present. The reflection and discussion among the researchers during analysis helped reduce the influence of individual researcher’s experiences and views on the interpretation.

This qualitative study was conducted during an RCT that had strict exclusion criteria, and some of the GPs’ experiences of care managers can be explained in part by this study design. GPs could only send patients with mild to moderate depression to care managers. This limitation contributed to the experiences expressed in the category *Care managers should be assigned to patients who need them the most*. After the end of the RCT, the care managers’ role was changed. Care managers now also work with patients who have severe and/or persistent mental health problems.

## Conclusions

GPs have varied views and experiences of care managers. As a complementary part of the primary health care team, care managers can be useful for patients with depression, but team members’ roles must be clear.
